# Observations on Intermittent Fevers

**Published:** 1809-06

**Authors:** 


Medical and Physical Journal.

VOL. XXI.] JUNE, 1809. [NO. 124.

*Printed for R. PHILLIPS, by W. Thorne, Red Lion Court, Fleet Street, London.*

### *To the Editors of the Medical and Physical Journal.*

GENTLEMEN,

THE great prevalence of intermittent fevers in many extensive districts in this kingdom, has excited an uncommon share of the attention of the medical practitioner in those situations. This disease most commonly occurs among the poor, and owing to the great price of cinchona, medical men are obliged to have recourse to other remedies, the principal of which is arsenic; and there are many practitioners who give this medicine a decided preference to cinchona. It certainly possesses one considerable recommendation, that it may be given in a more eligible form; and from its being almost tasteless, it is not so liable to be refused by infants and children. From the active nature of this mineral, its exhibition ought to be attended with great care. And from this consideration I am induced to offer you these observations, having seen several cases where mischief has followed from its being used too freely, and expecting to see many more, as it is become extremely common for "Lady Doctors,"* and other persons who are not in the profession, to dispense the arsenic solution with a very liberal hand to the poor and needy.

During the last two years I have had numberless opportunities of noticing it effects upon a variety of constitutions, and persons of all ages. It is a medicine, upon which I place great reliance, and if properly managed, may be given with perfect safety. But, as I said before, the disease occurs generally among the poor, and persons

* Only three days since, I heard a gentleman say, that upon calling upon a female acquaintance, he found her busy in dispensing arsenic; and during his stay, which was but short, she had eleven patients; each receiving in their turn, a bottle of the mineral solution.

in low situations of life, where you cannot always insure a regular attention to the exhibition of any medicine; and thus the failure of this medicine in affecting a cure, is often attributable to this cause. I usually trust a poor person with eight doses of the solution, mixed with any common tincture, or with mint water, in an ounce phial, ordering the dose to be taken every six hours; the dose to be increased or diminished according to the age of the patient. For an adult I order from six to eight drops. If this quantity is taken without altering the paroxysm, or producing any ill effects, I give the patient eight doses more; and if this quantity does not effect a cure, I always think it proper to discontinue a repetition of it for two days at least, after which time it may be recommenced as before. It may be necessary to state, that I usually give an emetic, and a calomel or some other purge, before the arsenic; and during its exhibition, any other medicine that may be indicated by particular symptoms.

When this mineral exhibits its deleterious effect upon the constitution from small and repeated doses, which I think ought rarely to happen, the symptoms, as far as I have observed, are nausea and sickness; pain in the stomach; loss of appetite; tremor; coldness of the extremities; and sometimes diarrhœa comes on. If the medicine be persisted in, these symptoms will be increased, and may become alarming; but I have never known any unpleasant results from these symptoms, if upon their first appearance the medicine was discontinued. But perhaps it may be proper to give a purgative, followed by some cordial medicine.

I feel no hesitation in declaring, that in proper hands, arsenic will always prove a safe and efficacious medicine, and perhaps will as seldom disappoint a medical man as cinchona.

I have been surprised to hear a medical lecturer to a numerous class of pupils in London, reprobate the exhibition of arsenic in strong terms. He certainly mentioned several cases, where it had proved extremely injurious. But this I suspect must have happened from mismanagement. This Gentleman does not speak from his own experience or from his known accuracy and precision, he would find no reason to condemn a cautious use of arsenic in other diseases as well as in agues. I particularly mention this, as the opinion of a Lecturer in such a situation is likely to bias a numerous and respectable class of pupils. I am, &c.

*May* 8, 180[illegible]. AN ESSEX PRACTITIONER.

